# Efficacy of Negative Pressure Suction‐Assisted Flexible Ureteroscopy for Upper Urinary Tract Calculi and Construction of a Stone‐Free Rate Prediction Model

**DOI:** 10.1002/kjm2.70299

**Published:** 2026-10-12

**Authors:** Ren‐Hui Qiu, Ding‐Sheng He, Chun‐Yu Guo, Li Dong, Guo‐Zhang Lin

**Affiliations:** ^1^ Department of Urology 900th Hospital of PLA Joint Logistic Support Force Fuzhou China; ^2^ Department of Prevention Fuzhou Center for Disease Control and Prevention Fuzhou China

**Keywords:** flexible negative‐pressure suction sheath, flexible ureteroscopic lithotripsy, predictive model, stone‐free rate, upper urinary tract stones

## Abstract

This study retrospectively analyzed 92 patients who underwent negative‐pressure suction sheath‐assisted flexible ureteroscopic lithotripsy (observation group) and 85 patients who underwent conventional flexible ureteroscopic lithotripsy (control group). Propensity score full matching and weighted analyses were used to balance covariates. Operative time, hospital stay, pain scores, hemoglobin changes, white blood cell counts, neutrophil counts, stone clearance rates, and complications were compared, and predictive models for stone clearance were constructed. The observation group achieved significantly higher stone‐free rates at 3 days (69.6% vs. 29.7%, *p* < 0.001) and 1 month (75.0% vs. 34.2%, *p* < 0.001) postoperatively. Relative to the control group, the observation group had postoperative white blood cell counts closer to normal values (9.25 ± 3.26 vs. 10.08 ± 3.78, *p* < 0.05), with a smaller increase from baseline (0.82 ± 4.90 vs. 2.96 ± 5.02, *p* < 0.05). The neutrophil percentage was also lower (0.73 ± 0.10 vs. 0.77 ± 0.07, *p* < 0.05), and visual analogue scale pain score was significantly reduced (2.00 ± 0.53 vs. 2.56 ± 0.89, *p* < 0.001). The predictive models achieved areas under the receiver operating characteristic curve of 0.727 (95% confidence interval: 0.616–0.839) at 3 days and 0.742 (95% confidence interval: 0.630–0.855) at 1 month postoperatively, indicating good discriminatory ability. This study shows that flexible negative‐pressure suction sheath‐assisted flexible ureteroscopic lithotripsy improves short‐ and mid‐term stone clearance rates in patients with upper urinary tract stones. The models perform well and may facilitate individualized clinical decision‐making.

**Abbreviations**
AUC: area under the receiver operating characteristic curve
CI: confidence interval
DCA: decision curve analysis
FANS: flexible and navigable suction ureteral access sheath
FURS: flexible ureteroscopy
ISFR: immediate stone‐free rates
OR: odds ratio
PSM: propensity score matching
RR: relative risk
SFR: the stone‐free rate
SIRS: systemic inflammatory response syndrome
SMD: standardized mean differences
VAS: visual analogue scale

## 1 Introduction

Urolithiasis is a common global urological disorder that imposes a substantial public health burden. A 2025 systematic review of 1,276,826 participants reported a global prevalence of approximately 10.85% with marked regional heterogeneity [1], With advances in minimally invasive urological surgery, flexible ureteroscopy (FURS) has emerged as a first‐line treatment for upper urinary tract stones. Compared with extracorporeal shock wave lithotripsy, FURS achieves higher stone‐free rates (SFRs) and lower retreatment rates [2] Relative to percutaneous nephrolithotomy, FURS is associated with less surgical trauma, lower bleeding risk, and shorter hospital stays, enabling its widespread use for renal calculi ≤ 2 cm [3].

Nevertheless, conventional FURS has inherent limitations in achieving optimal stone clearance. Intraoperative lithotripsy primarily fragments stones, whereas small fragments depend on passive postoperative excretion, which results in a suboptimal immediate SFR [4]. Residual fragments increase the risks of renal colic, ureteral obstruction, secondary infection, and severe complications, including systemic inflammatory response syndrome and sepsis [5]. Notably, infection stones, which account for 15% of urinary calculi, show rapid growth, staghorn formation, high recurrence rates, and severe infectious complications, further underscoring the shortcomings of conventional lithotripsy [6]. Therefore, improving stone clearance while reducing intrarenal pressure and infection‐associated complications remains a critical clinical need.

The flexible and navigable suction ureteral access sheath (FANS) effectively addresses these clinical challenges. This device integrates a surgical working channel with external negative‐pressure suction, enabling simultaneous lithotripsy and stone fragment extraction [7]. Multiple meta‐analyses have reported the superior clinical efficacy for FANS. A recent systematic review and meta‐analysis found that FANS doubled postoperative day‐1 SFR (RR = 2.12) and reduced postoperative fever risk by 67% (RR = 0.33) compared with conventional sheaths [8]. A 2025 meta‐analysis similarly reported that FANS significantly improved 30‐day SFR (OR = 2.37) and decreased postoperative fever incidence by 69% (OR = 0.31) [9]. FANS also achieved better immediate and long‐term stone‐free outcomes than conventional FURS for 1–2 cm renal stones [10].

Although existing evidence preliminarily supports the clinical value of FANS, most studies have mainly focused on short‐term outcomes, and systematic evaluations of postoperative inflammatory responses, pain control, and their dynamic changes remain limited. Research on individualized SFR prediction models remains at an early stage. This retrospective study therefore compared the clinical outcomes of flexible negative‐pressure suction sheath‐assisted flexible ureteroscopic lithotripsy with conventional flexible ureteroscopic lithotripsy for upper urinary tract stones. The study focused on operative time, hospital stay, pain scores, hemoglobin changes, inflammatory markers, stone clearance rates, and complications and aimed to construct an SFR prediction model to provide evidence‐based support for clinical decision‐making.

## 2 Materials and Methods

### 2.1 Study Population and Patient Selection

This single‐center retrospective cohort study with a non‐concurrent control group included 177 patients with upper urinary tract stones treated at our hospital. The observation group comprised 92 patients who underwent flexible ureteroscopic lithotripsy assisted by a negative‐pressure suction sheath between August 2024 and July 2025, whereas the control group comprised 85 patients who received conventional flexible ureteroscopic lithotripsy between January and June 2023. Because treatment allocation was perfectly collinear with operative time, a treatment‐time interaction model could not be established. *E*‐value sensitivity analysis was performed for SFR to assess the potential impact of unmeasured temporal confounders, including improved surgical proficiency, equipment updates, and evolving perioperative protocols. An additional 80 patients were recruited for temporal external validation: 40 suction sheath‐assisted cases from July 2023 to June 2024 and 40 conventional cases from January to December 2022. Eligible patients were aged ≥ 18 years, had imaging‐confirmed upper urinary tract stones (renal or proximal ureteral stones), and underwent flexible ureteroscopic holmium laser lithotripsy performed by the same surgical team using the same instruments. Complete perioperative imaging and follow‐up data were also required. Exclusion criteria included severe preoperative renal dysfunction, previous urinary tract surgery, severe cardiopulmonary or coagulation disorders, contraindications to endoscopic surgery, pregnancy, ureteral strictures or severe tortuosity preventing stone access, congenital urinary tract malformations, preoperative fever or systemic inflammation, and incomplete clinical or imaging data. During 2022–2025, surgical teams, core equipment, preoperative CT criteria, follow‐up schedules, and perioperative anti‐infective and analgesic protocols remained unchanged, limiting time‐dependent differences. This study complied with the Declaration of Helsinki and subsequent amendments.

### 2.2 Treatment Procedures

All patients underwent routine urinalysis and midstream urine culture preoperatively. Those with positive cultures received targeted antibiotic therapy, whereas those with negative cultures received prophylactic antibiotics according to standard clinical practice.

#### 2.2.1 Observation Group

After general anesthesia, patients were placed in the lithotomy position, and the perineal area was disinfected and draped. A ureteroscope was inserted transurethrally to examine the bladder and identify the affected ureteral orifice. Under guidewire guidance, the ureteroscope was advanced to the proximal ureter for anatomical assessment, after which the guidewire was retained and the ureteroscope withdrawn. A flexible ureteroscopic access sheath (11/12 Fr, Zhejiang Medical High‐tech Co. Ltd.) was inserted over the guidewire. The negative‐pressure suction tube was connected to the sheath side port and attached to a suction device, followed by insertion of the flexible ureteroscope through the sheath (Figure 1A,B). Stone fragmentation was performed using a 200 μm holmium laser fiber at preset parameters (e.g., 1.4 J at 20 Hz). Irrigation flow was maintained at approximately 0.4 L/h, with a preset irrigation pressure of 400 mmHg and an actual intraluminal pressure of approximately 200 mmHg. Negative‐pressure suction was adjusted between −60 and −80 mmHg to maintain low intrarenal pressure and facilitate timely stone fragment removal. After the procedure, the sheath and ureteroscope were removed, and a ureteral stent (Zhejiang Medical High‐tech Co. Ltd.) was placed.

**FIGURE 1:**
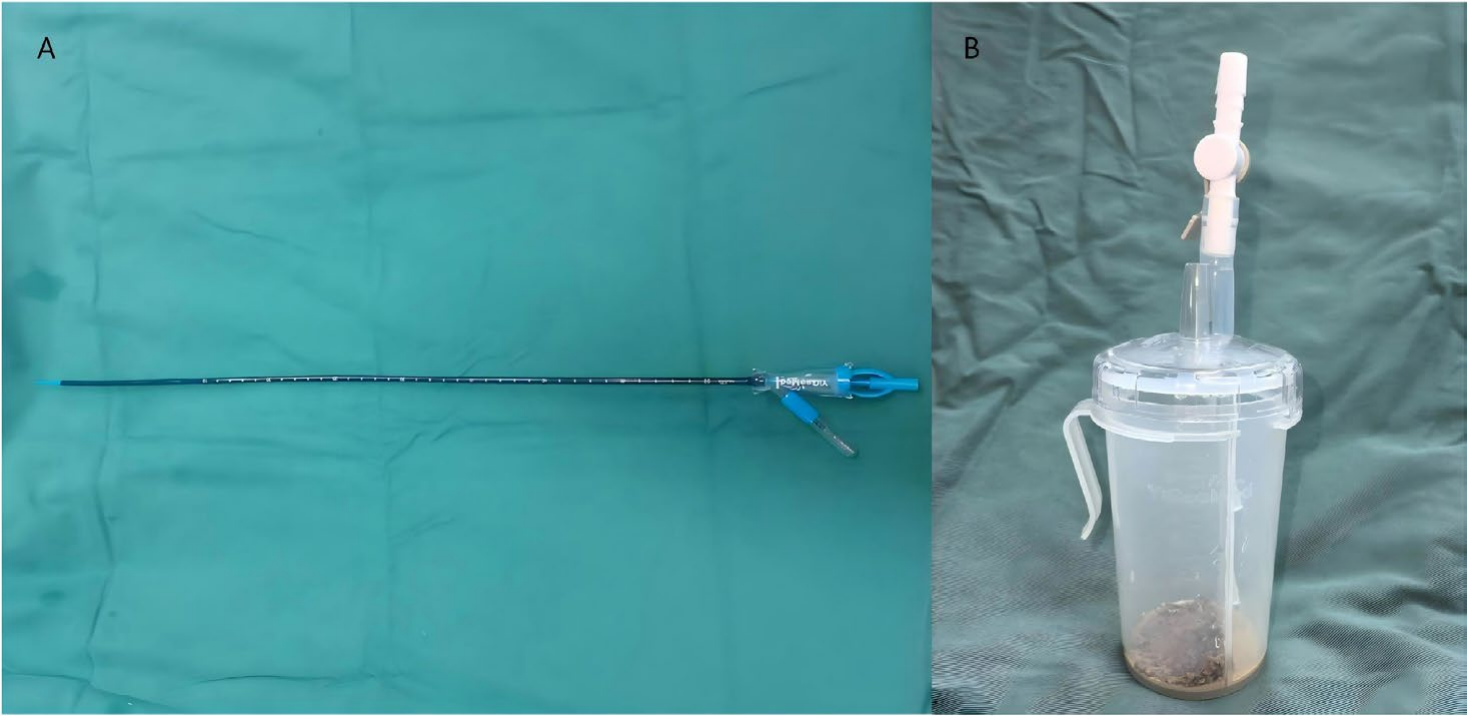
Flexible ureteroscopic lithotripsy assisted by a negative‐pressure suction sheath. (A) Curved negative‐pressure suction sheath; (B) Negative‐pressure suction cup.

#### 2.2.2 Control Group

The control‐group procedure was identical to that in the observation group, except that no suction was applied. Larger fragments were retrieved using a stone extraction basket, whereas smaller fragments were allowed to pass spontaneously after surgery.

In general, residual fragments < 4 mm were considered clinically insignificant after flexible ureteroscopic lithotripsy. All ureteral access sheaths and postoperative ureteral stents used throughout the cohort were from the same manufacturer to minimize potential confounding from differences in equipment brands.

### 2.3 Development and Validation of Predictive Nomogram Models

Multivariable logistic regression models were constructed using the lrm function of the rms package, incorporating all clinically relevant covariates to identify independent predictors of early and 1‐month stone clearance. Following the TRIPOD statement, a separate, non‐overlapping cohort recruited at the same center during a different period was defined as the temporal external validation cohort. This approach evaluates reproducibility across clinical periods and differs from random internal split‐sample and multicenter geographic external validation. Temporal external validation assessed three key aspects of model performance: discrimination, evaluated by the area under the receiver operating characteristic curve (AUC) with 95% confidence intervals (CIs); calibration, assessed using calibration curves generated by the val.prob. function, with the calibration intercept, slope, Brier score, and Hosmer‐Lemeshow statistic; and clinical utility, evaluated by decision curve analysis to determine net clinical benefit across threshold probabilities.

### 2.4 Statistical Analysis

Statistical analyses were performed using R 4.3.1. For pre‐matching baseline comparisons, continuous‐variable normality was assessed with the Shapiro–Wilk test on raw data; normally distributed variables were compared using Welch‐corrected Student's *t*‐test, and non‐normally distributed variables using the Wilcoxon rank‐sum test. Categorical variables were analyzed using the chi‐squared test, or Fisher's exact test when any expected cell count was < 5. Propensity score full matching was performed, and matching weights were applied to all post‐matching comparisons. Normality was then evaluated from weighted regression residuals. Weighted *t*‐tests and weighted Wilcoxon rank‐sum tests were used for normally and non‐normally distributed continuous variables, respectively, and weighted chi‐squared tests for categorical variables. For non‐normally distributed postoperative continuous outcomes, 95% CIs of weighted median differences (observation minus control) were generated from 1000 bootstrap resamples with replacement. Standardized mean differences (SMDs) quantified covariate balance before and after matching. Postoperative outcomes were analyzed using weighted tests based on residual normality. Weighted logistic regression computed ORs and 95% CIs for binary endpoints. Multivariable weighted logistic regression identified independent predictors of surgical outcomes. *E*‐value sensitivity analysis assessed bias from unmeasured confounders for the primary stone‐free outcome. All tests were two sided, with *p* < 0.05 considered statistically significant.

## 3 Results

### 3.1 Propensity Score Matching (PSM)

Before matching, age (*p* = 0.026, SMD = 0.338) and stone location (*p* = 0.004, SMD = 0.519) differed significantly between the groups, indicating baseline imbalance (Table 1, Figure 2A–L). Propensity score full matching was applied to reduce selection bias while retaining all eligible patients. After matching, the effective sample sizes were 83 in the control group and 92 in the observation group (Table 2). No baseline covariate differed significantly between the groups (all *p* > 0.05). Absolute SMDs ranged from 0.007 to 0.204, and only stone diameter slightly exceeded the accepted threshold of 0.2, indicating acceptable covariate balance.

**TABLE 1:** Comparison of baseline characteristics between the control and observation groups.

Characteristics	Level	Control (*n* = 85)	Observation (*n* = 92)	*p*	SMD
Age/years		49.25 (12.67)	53.40 (11.94)	0.026	0.338
BMI/(kg/m^2^)		24.76 (3.29)	24.82 (3.91)	0.914	0.016
Stone_number/*n*		2.00 (1.00)	1.96 (1.03)	0.776	0.043
Diameter/cm		2.52 (0.88)	2.55 (0.93)	0.873	0.024
CT value/HU		1060.98 (252.92)	1001.01 (305.14)	0.158	0.214
Preoperative_creatinine/(μmol/L)		104.65 (76.42)	107.52 (95.39)	0.826	0.033
Preoperative_white_blood_cell_count/(×10^9^/L)		8.62 (4.20)	8.42 (4.32)	0.757	0.047
Preoperative_neutrophil_percentage (%)		0.67 (0.11)	0.68 (0.12)	0.600	0.079
Gender (%)	Female	29 (34.1)	36 (39.1)	0.593	0.104
	Male	56 (65.9)	56 (60.9)		
Location (%)	Renal stone	10 (11.8)	30 (32.6)	0.004	0.519
	Renal and ureteral stones	38 (44.7)	32 (34.8)		
	Ureteral stone	37 (43.5)	30 (32.6)		
Direction (%)	Left	45 (52.9)	49 (53.3)	1.000	0.006
	Right	40 (47.1)	43 (46.7)		
Severity_of_hydronephrosis (%)	Mild	53 (62.4)	59 (64.1)	0.673	0.187
	Moderate	8 (9.4)	12 (13.0)		
	None	8 (9.4)	5 (5.4)		
	Severe	16 (18.8)	16 (17.4)		

*Note:* Categorical variables are presented as *n* (%). Normally distributed continuous variables are reported as mean ± standard deviation, and non‐normally distributed continuous variables are expressed as median (interquartile range). Normality was evaluated using the Shapiro–Wilk test on raw observed data. For statistical comparisons, the chi‐squared test was used for categorical variables with expected cell counts ≥ 5, and Fisher's exact test was applied when any expected cell count was < 5. Normally‐distributed continuous variables were compared using Student's *t*‐test with Welch's correction, whereas non‐normally distributed continuous variables were analyzed with the Wilcoxon rank‐sum test. Standardized mean difference (SMD) was calculated to quantify intergroup covariate imbalance before propensity score matching.

Abbreviations: BMI, body mass index; CT, computed tomography; HU, Hounsfield unit.

**FIGURE 2:**
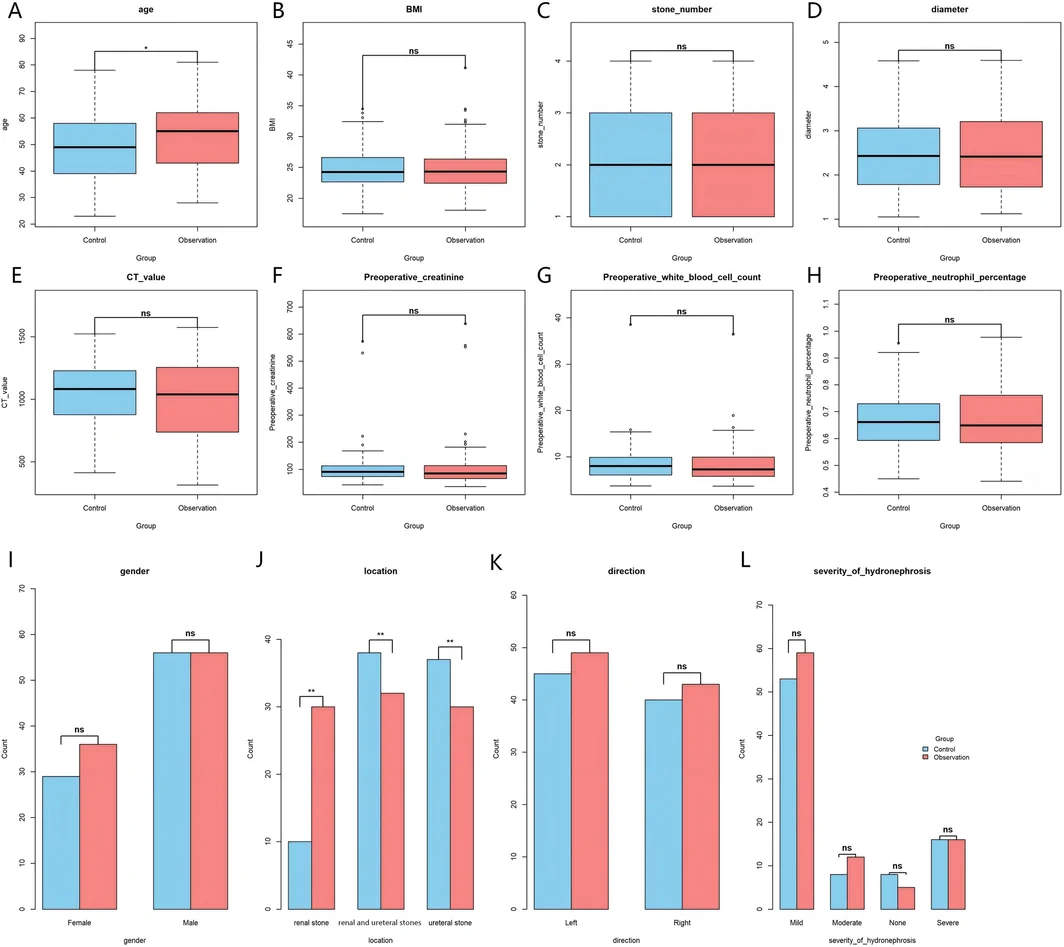
Comparison of baseline characteristics between the control and observation groups before propensity score matching (PSM). Panels (A–H) display boxplots of continuous variables, whereas panels (I–L) present bar charts of categorical variables. (A) Age; (B) Body mass index (BMI); (C) Stone number; (D) Maximum stone diameter; (E) Stone CT attenuation value; (F) Preoperative serum creatinine; (G) Preoperative white blood cell count; (H) Preoperative neutrophil percentage; (I) Gender; (J) Stone anatomical location; (K) Stone lateral direction; (L) Hydronephrosis severity. Normality of continuous variables was assessed via the Shapiro–Wilk test on raw data. Normally distributed continuous variables were compared using the independent Student's *t*‐test with Welch's correction, whereas non‐normally distributed variables were analyzed using the Wilcoxon rank‐sum test. Categorical variables were compared using the chi‐squared test when all expected cell counts were ≥ 5; Fisher's exact test was applied when any expected cell count was < 5. *p < 0.05, **p < 0.01; ns, non‐significant difference (*p* > 0.05).

**TABLE 2:** Comparison of baseline characteristics between the control and observation groups after PSM.

Characteristics	Level	Control (*n* = 83)	Observation (*n* = 92)	*p*	SMD
Age/years		55.32 ± 11.98	53.40 ± 11.94	0.371	−0.161
BMI/(kg/m^2^)		24.0 (22.0–26.3)	24.2 (22.4–26.3)	0.808	0.060
Stone_number/*n*		2 (1–3)	2 (1–3)	0.566	−0.091
Diameter/cm		2.6 (2.1–3.3)	2.4 (1.7–3.2)	0.264	−0.204
CT value/HU		1049 (859–1210)	1038 (688–1250)	0.429	−0.132
Preoperative_creatinine/(μmol/L)		90.8 (70.7–117.2)	84.0 (65.0–113.0)	0.511	0.023
Preoperative_white_blood_cell_count/(×10^9^/L)		7.67 (6.25–9.99)	7.28 (5.79–9.69)	0.668	−0.029
Preoperative_neutrophil_percentage (%)		0.68 (0.60–0.75)	0.65 (0.58–0.76)	0.947	−0.007
Gender (%)	Female	32 (38.0)	36 (39.1)	0.901	0.024
	Male	51 (62.0)	56 (60.9)		
Location/*n* (%)	Renal stone	29 (34.8)	30 (32.6)	0.967	−0.046
	Renal and ureteral stones	28 (33.9)	32 (34.8)		
	Ureteral stone	26 (31.3)	30 (32.6)		
Direction (%)	Left	45 (53.8)	49 (53.3)	0.957	−0.010
	Right	38 (46.2)	43 (46.7)		
Severity_of_hydronephrosis/*n* (%)	None	6 (6.7)	5 (5.4)	0.970	0.080
	Mild	50 (60.3)	59 (64.1)		
	Moderate	11 (12.8)	12 (13.0)		
	Severe	17 (20.2)	16 (17.4)		

*Note:* Normality of continuous variables was evaluated using the Shapiro–Wilk test on weighted regression residuals. Normally distributed continuous variables are presented as mean ± standard deviation and compared using a weighted *t*‐test. Non‐normally distributed continuous variables are reported as median (interquartile range) and compared using the weighted Wilcoxon rank‐sum test. Categorical variables are shown as *n* (%) and compared using the weighted chi‐squared test. Standardized mean difference (SMD) was used to evaluate covariate balance after propensity‐score full matching; SMD < 0.1 indicates excellent balance, and SMD < 0.2 represents satisfactory balance.

Abbreviations: BMI, body mass index; HU, Hounsfield unit.

*E*‐value sensitivity analysis assessed robustness against unmeasured temporal confounding. For 3‐day SFR, the point‐estimate *E*‐value was 2.33 and the lower‐bound *E*‐value was 4.09; for 1‐month SFR, the corresponding values were 2.40 and 4.24. These findings indicated that only strong unmeasured temporal confounders could negate the observed benefit of suction sheath‐assisted lithotripsy, and minor improvements in clinical practice were unlikely to alter the primary conclusions (Table S1).

### 3.2 Comparison of Postoperative Outcomes

All postoperative outcome analyses were weighted by matching scores (Table 3). The observation group achieved markedly higher 3‐day (69.6% vs. 29.7%; OR = 5.42, 95% CI: 2.39–12.31, *p* < 0.001) and 1‐month SFR (75.0% vs. 34.2%; OR = 5.77, 95% CI: 2.58–12.89, *p* < 0.001). It also showed lower postoperative white blood cell counts, smaller increases in inflammatory cell levels, lower neutrophil percentages, and reduced visual analogue scale (VAS) pain scores (all *p* < 0.001). Operative time, hospital stay, postoperative creatinine, hemoglobin decrease, and fever rate did not differ between the groups (all *p* > 0.05; fever incidence: 1.1% vs. 1.8%, *p* = 0.668).

**TABLE 3:** Comparison of perioperative and postoperative outcomes between the control and observation groups after propensity score full matching.

Outcomes	Control (*n* = 83)	Observation (*n* = 92)	*Z*/OR	*p*	95% CI
Duration_of_surgery/minutes	80 (51–167)	80 (52–110)	−1.00	0.319	(−43.05, 20.00)
Length_of_hospital_stay/days	7 (6–8)	6 (5–8)	−1.76	0.080	(−1.00, 0.00)
Postoperative_length_of_stay/days	4 (4–4)	4 (4–4)	−0.42	0.673	(0.00, 0.00)
Postoperative_creatinine/(μmol/L)	88.8 (68.3–109.3)	80.0 (64.0–97.0)	−1.26	0.208	(−20.20, 7.61)
Decrease_in_hemoglobin/(g/L)	9 (4–13)	7 (4–11)	−0.74	0.462	(−4.00, 1.00)
Postoperative_white_blood_cell_count/(×10^9^/L)	10.71 (8.90–11.89)	8.55 (7.29–10.63)	−3.04	0.003	(−3.40, −0.33)
Postoperative_neutrophil_percentage (%)	0.78 (0.71–0.82)	0.75 (0.69–0.79)	−2.32	0.022	(−0.06, −0.01)
Change_in_white_blood_cell_count/(×10^9^/L)	3.34 (1.46–5.58)	0.71 (−0.75–3.32)	−3.78	< 0.001	(−3.46, −1.36)
VAS score/(points)	3 (2–3)	2 (2–2)	−3.84	< 0.001	(−1.00, 0.00)
Fever (≥ 38.5°C) (%)	1 (1.8)	1 (1.1)	0.61	0.668	(0.07, 5.79)
SFR_at_postoperative_day_3 (%)	25 (29.7)	64 (69.6)	5.42	< 0.001	(2.39, 12.31)
SFR_at_postoperative_month_1 (%)	28 (34.2)	69 (75.0)	5.77	< 0.001	(2.58, 12.89)

*Note:* Continuous outcomes are presented as median (interquartile range). Comparisons of continuous variables were performed using the weighted Wilcoxon rank‐sum test; *Z* values are reported. The 95% confidence intervals for the weighted median difference (observation group minus control group) were derived from 1000 bootstrap resamples with replacement. Categorical outcomes are reported as *n* (%). Weighted logistic regression was used for binary endpoints to calculate odds ratios (ORs) and corresponding 95% confidence intervals. *p* < 0.05 was considered statistically significant.

Abbreviation: VAS, visual analogue scale.

### 3.3 Construction and Validation of Predictive Models for Postoperative SFR

Separate nomograms were developed to predict 3‐day and 1‐month SFR, incorporating treatment type, age, BMI, stone number, maximum stone diameter, stone CT value, hydronephrosis grade, operative time, hemoglobin loss, and stone location (Figure 3A,B). Total point scores were converted into individualized probabilities of stone clearance.

**FIGURE 3:**
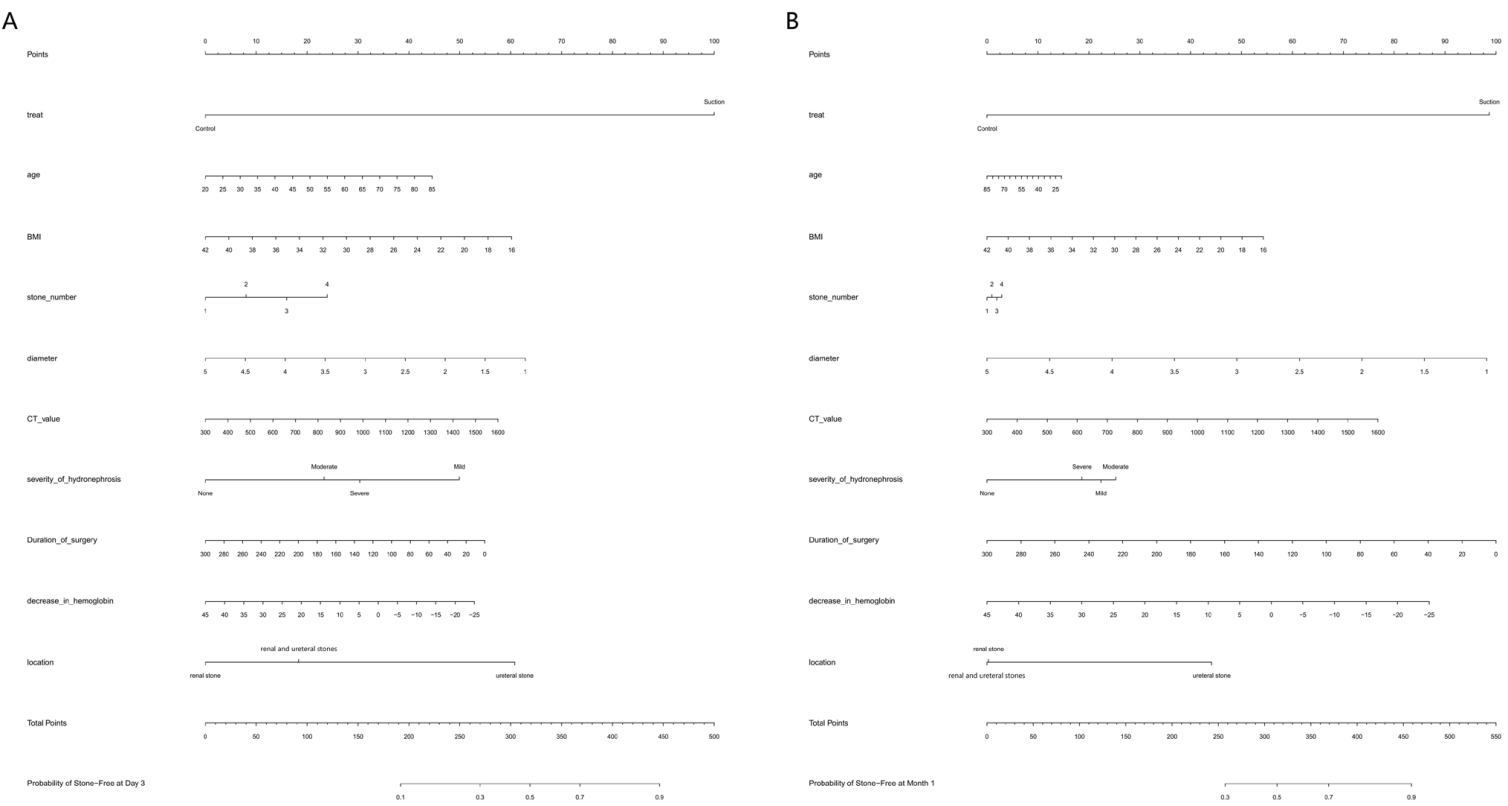
Nomogram prediction models for stone‐free rate (SFR) after flexible ureteroscopic lithotripsy constructed using the propensity score full‐matched training cohort. (A) Nomogram predicting the probability of achieving stone‐free status 3 days after surgery; (B) Nomogram predicting the probability of achieving stone‐free status 1 month after surgery. Each clinical covariate corresponds to an individual point value on the top point axis. The total point score, calculated by summing the points assigned to all variables, is matched to the bottom axis to obtain the individualized predicted probability of complete stone clearance. Predictive variables included treatment modality, age, body mass index (BMI), stone number, maximum stone diameter, stone CT attenuation value, hydronephrosis severity, operative duration, hemoglobin decline, and stone anatomical location. SFR, stone‐free rate; BMI, body mass index.

Temporal external validation evaluated model discrimination, calibration, and clinical utility. ROC analysis indicated moderate discrimination, with AUCs of 0.727 (95% CI: 0.616–0.839) for the 3‐day model and 0.742 (95% CI: 0.630–0.855) for the 1‐month model (Figure 4A,B). Calibration curves, low Brier scores, and small Emax values indicated good agreement between predicted and observed SFRs (Figure 4C,D). Decision curve analysis showed that both nomograms provided greater net clinical benefit than treat‐all or treat‐none strategies across a wide range of threshold probabilities (Figure 5). Overall, both nomograms offered reliable discrimination, favorable calibration, and clinical utility for individualized risk stratification in patients with upper urinary tract stones.

**FIGURE 4:**
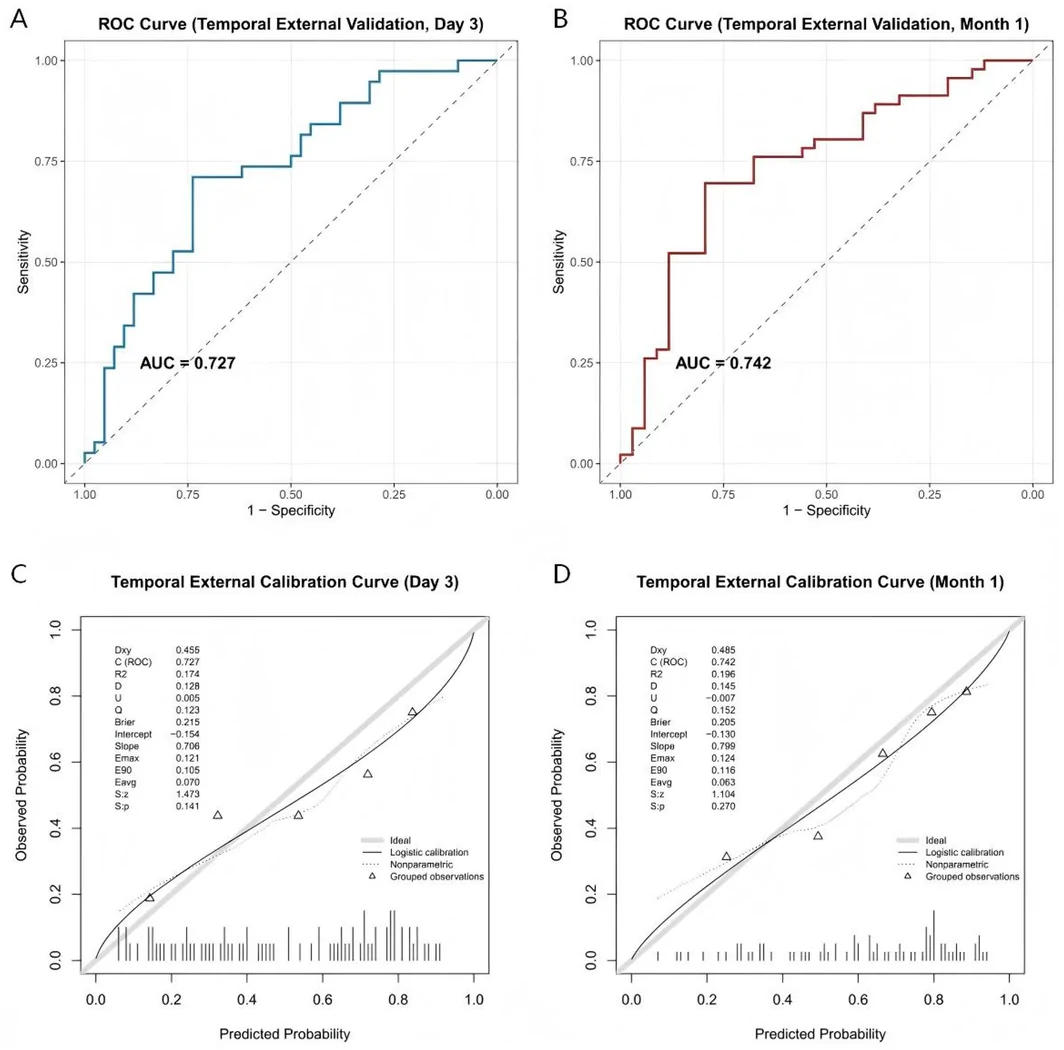
Temporal external validation of two nomogram models for predicting stone‐free rate (SFR) after flexible ureteroscopic lithotripsy in an independent validation cohort. (A) Receiver operating characteristic (ROC) curve of the prediction model for stone‐free status 3 days after surgery, with an area under the curve (AUC) of 0.727. The dashed diagonal line represents the reference line indicating no discrimination. (B) ROC curve of the prediction model for stone‐free status 1 month after surgery, with an AUC of 0.742. (C) Temporal external calibration curve of the day 3 nomogram. The gray solid line indicates ideal calibration; the black solid line represents the logistic calibration curve; triangular markers indicate grouped observed patients, and the histogram at the bottom displays the distribution of predicted probabilities in the validation cohort. (D) Temporal external calibration curve of the month 1 nomogram, with the same graphical definitions as those described in panel C. AUC, area under the receiver operating characteristic curve; ROC, receiver operating characteristic; SFR, stone‐free rate.

**FIGURE 5:**
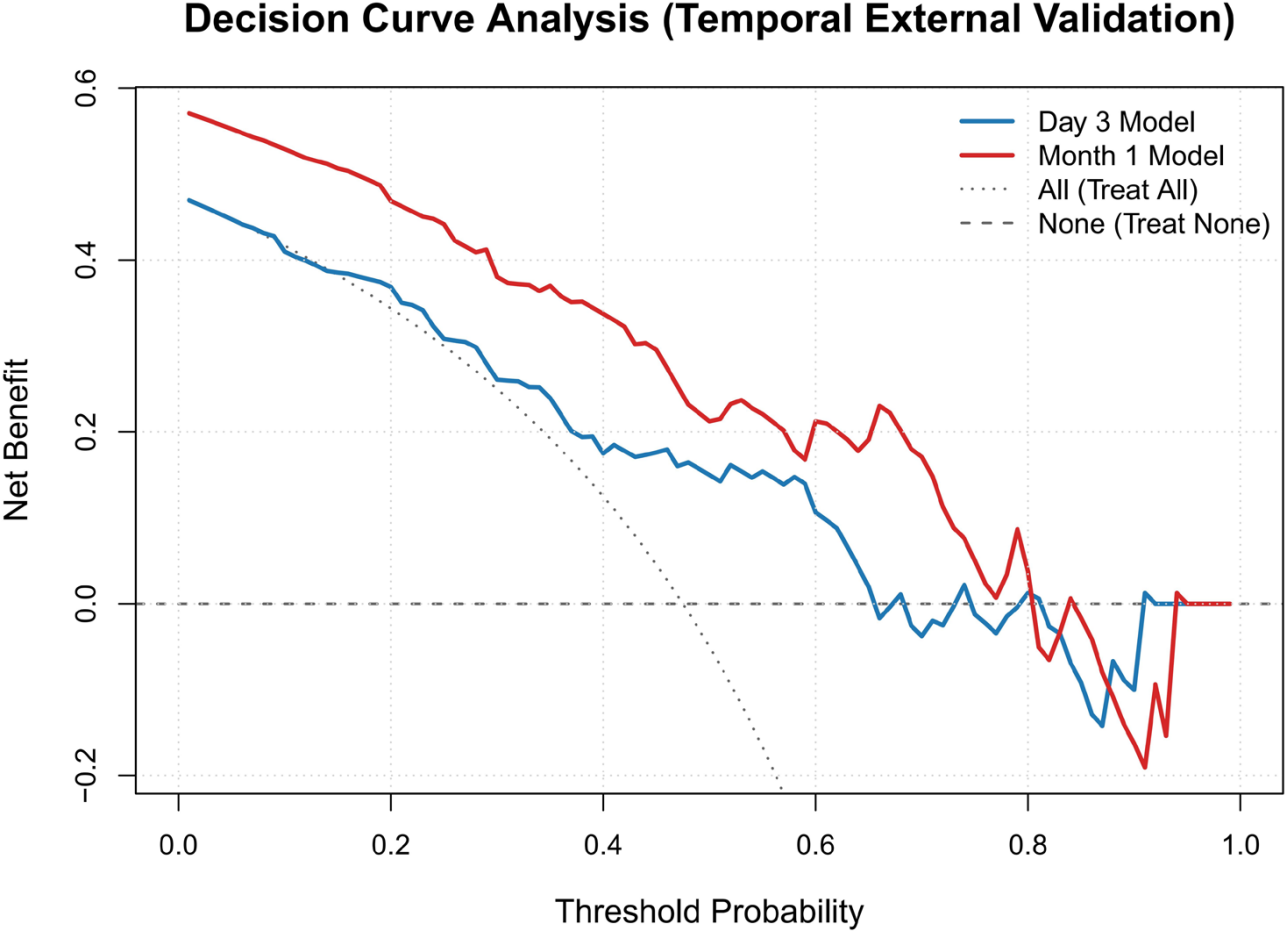
Decision curve analysis (DCA) of the two stone‐free rate (SFR) prediction models in the independent temporal external validation cohort. The blue solid curve represents the net clinical benefit of the nomogram predicting SFR 3 days after surgery, whereas the red solid curve represents the nomogram predicting SFR 1 month after surgery. The gray dotted line denotes the treat‐all strategy, and the gray dashed horizontal line represents the treat‐none strategy. The *X*‐axis shows the threshold probability, and the *Y*‐axis indicates the standardized net clinical benefit. DCA, decision curve analysis; SFR, stone‐free rate.

## 4 Discussion

This retrospective study used propensity score full matching to balance baseline covariates and weighted statistical analyses for intergroup comparisons, enabling a systematic comparison of negative‐pressure suction sheath‐assisted flexible ureteroscopic lithotripsy with conventional flexible ureteroscopic lithotripsy for upper urinary tract stones. The suction sheath‐assisted technique significantly improved 3‐day and 1‐month SFRs, reduced postoperative inflammatory responses, and alleviated postoperative pain.

Regarding stone clearance efficacy, the higher SFR observed in the observation group may be attributed to the unique intraoperative mechanism of the negative‐pressure suction system. Continuous intraoperative suction facilitates the rapid removal of stone debris and blood clots [11], reduces residual fragments, maintains low intrarenal pressure, and decreases fluid extravasation and bacterial backflow [12]. These advantages are consistent with previous clinical evidence. A prospective study of 226 patients reported higher early and long‐term SFRs in the suction sheath group (85.8% on postoperative day 1 and 98.2% at 1 month) than in the conventional sheath group (72.6% and 90.3%, respectively; all *p* < 0.05) [13]. A recent meta‐analysis further confirmed that suction sheath‐assisted FURS substantially improves stone clearance and reduces the risk of postoperative fever [14].

Regarding postoperative inflammation and pain, the suction sheath group showed milder inflammatory responses, which may be related to stable low intrarenal pressure during surgery [15]. Conventional ureteroscopy relies on passive drainage, causing fluctuating elevations in intrarenal pressure during repeated irrigation and lithotripsy. Such pressure increases may induce pyelovenous and pyelolymphatic backflow, facilitating systemic dissemination of inflammatory mediators and endotoxins and triggering inflammatory cascades [16]. In contrast, negative‐pressure suction enables active real‐time drainage of irrigation fluid, stone debris, and exudates, sustaining intrarenal pressure within a safe range (generally < 20 mmHg) and reducing pressure‐mediated inflammatory activation [17]. Animal studies have further shown that intermittent suction can rapidly reduce intrarenal pressure and lower peak intrarenal pressure by approximately 40% [18].

Additionally, lower VAS pain scores in the observation group were attributable to reduced mucosal irritation [19], decreased renal capsular distension [20], and efficient removal of residual stone fragments [21]. A multicenter prospective study of 540 patients supported these findings, reporting milder postoperative flank pain and shorter hospital stays after suction sheath‐assisted lithotripsy (*p* < 0.001) [22]. In terms of surgical trauma, both techniques caused only mild hemoglobin reductions, suggesting limited perioperative occult blood loss and ureteral mucosal injury.

For predictive model construction, this study established an SFR prediction model based on readily available clinical variables, including surgical modality, age, BMI, stone diameter, stone CT value, hydronephrosis severity, and operative duration. Previous studies have identified several risk factors associated with unsatisfactory stone clearance after suction‐assisted lithotripsy, such as multicalyceal stone location and elevated Guy's stone scores [23]. Unlike existing models, ours incorporated negative‐pressure suction sheath use as a key predictive variable and underwent temporal external validation, supporting improved generalizability. Despite moderate AUCs (0.7–0.8), the models achieved reliable discrimination using routine clinical indicators, without high‐dimensional radiomic or biomolecular data, supporting clinical accessibility and implementation.

With regard to safety, the negative‐pressure suction technique did not increase postoperative complications and showed favorable inflammatory control, indicating a satisfactory safety profile. Active intraoperative suction may reduce repeated instrumentation and residual stone fragments, thereby lowering the risks of ureteral mucosal injury and subsequent stricture. A 2025 clinical study supported the safety of this technique, reporting a low short‐term postoperative fever rate (3.2%) and no cases of ureteral stricture [24]. Low and stable intrarenal pressure may also help prevent postoperative fever and bacteremia. A large‐sample propensity score‐matched study further showed that suction‐assisted lithotripsy reduced postoperative systemic inflammatory response syndrome while improving stone clearance [25]. Given the minimal intraoperative visible blood loss, postoperative hemoglobin reduction may serve as a sensitive indicator of subtle ureteral mucosal injury and microvascular bleeding [26]. The significantly smaller hemoglobin decrease in the observation group suggests that negative‐pressure suction lithotripsy may reduce ureteral mucosal trauma, contributing to its favorable minimally invasive profile.

Complete baseline indicators, including maximum stone diameter, total stone burden, stone number, stone location, and hydronephrosis grade, were documented for all participants to characterize stone complexity. Retrograde intrarenal surgery (RIRS) was performed in all patients in this study. According to the 2024 European Association of Urology Guidelines on Urolithiasis, percutaneous nephrolithotomy is recommended as the primary treatment for large renal stones [27], whereas FURS represents a minimally invasive alternative with broader indications [28, 29]. Unlike percutaneous nephrolithotomy, FURS is performed through the natural urinary tract without renal puncture or nephrostomy placement, thereby avoiding renal parenchymal trauma and major bleeding associated with the percutaneous tract [30]. The minimally invasive advantages of suction sheath‐assisted FURS are more pronounced in patients with solitary kidney [31], coagulation disorders, or elevated bleeding risks [32]. Additionally, FURS is associated with shorter hospital stays, less postoperative pain, and faster recovery, consistent with the minimally invasive treatment strategy adopted at our center [3, 33]. The present study further indicates that adding a negative‐pressure suction sheath to conventional flexible ureteroscopic lithotripsy provides additional clinical benefits.

This study has several methodological strengths. First, propensity score full matching balanced baseline covariates between groups, and weighted analyses were used for intergroup comparisons. This enhanced baseline comparability and reduced the selection bias inherent to retrospective studies. Second, the efficacy and safety of suction sheath‐assisted lithotripsy were evaluated across multiple aspects, including stone clearance, inflammatory responses, postoperative pain, and complications, providing multidimensional evidence for clinical decision‐making. Third, a validated prediction model for SFR was established to facilitate individualized risk assessment.

Several limitations of this study should be acknowledged. First, the single‐center, non‐concurrent retrospective design with non‐overlapping enrollment periods resulted in perfect collinearity between surgical modality and admission period, preventing any treatment‐time interaction or stratified analyses. Although propensity score full matching and weighted analyses were employed to enhance baseline comparability, and *E*‐value sensitivity analysis suggested robustness against temporal confounding, such adjustments cannot completely eliminate residual confounding. Because treatment allocation was intrinsically determined by the treatment period, unmeasured factors such as stone composition, antibiotic regimens, and intraoperative perfusion pressure may still have introduced bias. Temporal confounding therefore remains a concern and, together with the retrospective design, substantially limits the generalizability of our results. Multicenter external validation is urgently needed to confirm the reproducibility and applicability of our findings across different clinical settings and timeframes. Second, although a temporal external validation cohort was included, its modest sample size calls for confirmation in larger multicenter prospective studies. Third, the absence of radiomic or artificial intelligence‐based features indicates potential for model improvement. Fourth, the one‐month follow‐up is insufficient to evaluate long‐term outcomes. Future prospective multicenter studies with extended follow‐up are warranted to validate our results and comprehensively assess long‐term efficacy and safety.

## 5 Conclusion

This study shows that flexible negative‐pressure suction sheath‐assisted flexible ureteroscopic lithotripsy is superior to conventional flexible ureteroscopic lithotripsy in improving SFR and alleviating postoperative inflammation and pain, representing a safe and effective minimally invasive treatment option. The SFR prediction models based on this technique showed good discriminative ability and may facilitate individualized risk stratification and treatment planning. Given the single‐center retrospective design, multicenter prospective studies are needed to confirm long‐term efficacy. Future research should integrate radiomics and artificial intelligence algorithms to improve predictive accuracy and advance precision minimally invasive treatment for urolithiasis.

## Funding

This work was supported by the PLA Joint Logistic Support Force 900th Hospital Research Fund, 2022QC06.

## Ethics Statement

This retrospective study used de‐identified medical records and was approved by the Ethics Committee of the 900th Hospital of PLA Joint Logistic Support Force with a waiver of informed consent (Approval Number: 2026–114). All methods were carried out in accordance with relevant guidelines and regulations.

## Conflicts of Interest

The authors declare no conflicts of interest.

## Supporting information


**Table S1:** kjm270299‐sup‐0001‐Table S1.docx. *E*‐value analysis for the robustness of the association between treatment modality and postoperative stone‐free rate.

## Data Availability

The data that support the findings of this study are available from the corresponding author upon reasonable request.
