# Supplementary material for: Efficacy of Negative Pressure Suction‐Assisted Flexible Ureteroscopy for Upper Urinary Tract Calculi and Construction of a Stone‐Free Rate Prediction Model
Source: Kaohsiung J Med Sci. 2026 Oct 12:e70299. Online ahead of print. doi: 10.1002/kjm2.70299 (PMC13656727; doi:10.1002/kjm2.70299)
Supplement: Supplementary file 1 [file KJM2-9999-e70299-s001.docx]

| Outcome | OR | CI_Lower | CI_Upper | Evalue_Point | Evalue_CI_Lower |
| --- | --- | --- | --- | --- | --- |
| SFR_at_postoperative_day_3 | 5.418 | 5.368 | 5.469 | 2.33 | 4.09 |
| SFR_at_postoperative_month_1 | 5.767 | 5.715 | 5.82 | 2.4 | 4.24 |
